# Invasive coronary physiology: a Dutch tradition

**DOI:** 10.1007/s12471-020-01461-7

**Published:** 2020-08-11

**Authors:** T. P. van de Hoef, G. A. de Waard, M. Meuwissen, M. Voskuil, S. A. J. Chamuleau, N. van Royen, J. J. Piek

**Affiliations:** 1 Department of Clinical and Experimental Cardiology, Amsterdam UMC, location AMC, University of Amsterdam, Amsterdam, The Netherlands; 2 Department of Cardiology, Amphia Hospital, Breda, The Netherlands; 3 Department of Cardiology, University Medical Centre Utrecht, Utrecht, The Netherlands; 4 Department of Cardiology, Radboud University Medical Center, Nijmegen, The Netherlands

**Keywords:** Fractional flow reserve, Coronary flow reserve, Non-hyperaemic coronary pressure ratios, Stenosis resistance index, Microvascular resistance, Coronary flow capacity

## Abstract

Invasive coronary physiology has been applied since the early days of percutaneous transluminal coronary angioplasty, and has become a rapidly emerging field of research. Many physiology indices have been developed, tested in clinical studies, and are now applied in daily clinical practice. Recent clinical practice guidelines further support the use of advanced invasive physiology methods to optimise the diagnosis and treatment of patients with acute and chronic coronary syndromes. This article provides a succinct review of the history of invasive coronary physiology, the basic concepts of currently available physiological parameters, and will particularly highlight the Dutch contribution to this field of invasive coronary physiology.

## Dutch contribution to the field

- The development and clinical implementation of fractional flow reserve was driven by Professor Nico Pijls who, together with the team in Eindhoven, spent his whole career pursuing the broad clinical adoption of this invasive physiological tool.
- The same team developed and validated the coronary thermodilution technique for coronary flow and microvascular resistance assessment, and more recently introduced absolute flow and resistance measurements using this technique.
- For invasive coronary Doppler flow velocity measurements, Professor Jan Piek and the team in Amsterdam have driven both technical and clinical advances in its use in obstructive coronary artery disease, collateral flow, and non-obstructive coronary artery disease, and have governed the introduction of Doppler flow velocity-derived assessment of microvascular and stenosis resistance, as well as invasive assessment of coronary flow capacity.
- Dutch input also played an important role in the validation and clinical application of instantaneous wave-free ratio. As such, many of the physiological tools described in this review were influenced by Dutch investigators during their development, validation, or clinical implementation.

## Historical perspective

The presence of atherosclerotic narrowing disturbs the otherwise laminar flow inside a coronary artery. Friction generated by acceleration of flow at the throat of a coronary artery stenosis, and flow separation with the formation of eddies at the exit of the stenosis together lead to loss of kinetic energy identified by a reduction in perfusion pressure distal to the stenosis [1, 2]. Gruentzig already used the pressure gradient across a stenosis as a marker of stenosis severity, and its alleviation after balloon coronary angioplasty as a marker of procedural success [3]. These studies as well as the initial studies performed by Wijns et al. in the mid-1980s used the pressure gradient assessed through the balloon catheter [4]. However, since the size of a balloon catheter inevitably causes a pressure gradient across a lesion, its application for diagnostic purposes is cumbersome. As physicians embraced percutaneous transluminal coronary angioplasty for the treatment of coronary artery disease, overuse eventually became a problem as illustrated by the phrase ‘the oculo-stenotic reflex’ coined by Eric Topol [5]. To prevent overuse of angioplasty, tools available in the catheterisation laboratory to identify stenoses that in fact cause inducible myocardial ischaemia were needed. Consequently, coronary guidewires were developed that were equipped with either a pressure sensor or a Doppler flow velocity sensor to assess with high fidelity, for the first time in humans, the haemodynamic significance of coronary lesions [6–8]. This diagnostic armamentarium has since yielded a variety of physiological parameters that can be used to characterise the haemodynamic severity of a coronary stenosis, as well as the functional status of the coronary microcirculation. This review will describe the basic concepts of these parameters, and will particularly highlight the Dutch contribution to this field of invasive coronary physiology.

## Fractional flow reserve to measure functional stenosis severity

**Fig. 1:**
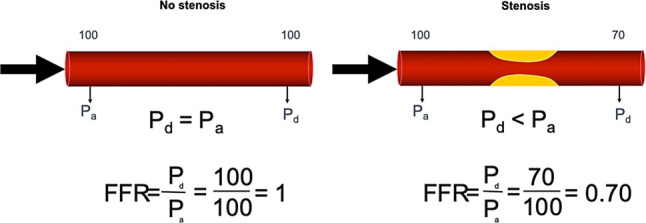
Concept of fractional flow reserve (FFR). FFR is defined as the ratio of mean proximal to mean distal coronary pressure. When no epicardial stenosis is present (*left panel*), the pressure loss across the coronary artery is negligible, and proximal aortic pressure (Pa) and distal coronary pressure (Pd) are equivalent, leading to an FFR of 1. In the presence of a stenosis (*right panel*), pressure loss across the stenosis will occur, and distal coronary pressure will be lower than proximal coronary pressure, leading to an FFR smaller than 1.0. In this example, the stenosis leads to a pressure gradient across the stenosis of 30 mm Hg, leading to an FFR of 0.70

In 1993, Pijls and colleagues proposed the fractional flow reserve (FFR) as a method to evaluate the functional severity of a stenosis [9]. FFR is defined as the ratio between mean distal coronary pressure and mean proximal coronary pressure, measured during maximal vasodilatation induced by a pharmacological agent such as adenosine. The FFR theorem depicts that, during hyperaemic conditions, a predictable relationship exists between distal coronary pressure and myocardial blood flow. As such, FFR describes the proportion of myocardial flow downstream of a coronary stenosis as a fraction of the theoretical maximal blood flow in that artery in the absence of the stenosis. For example, if the mean proximal pressure in the aorta is 100 mm Hg and the pressure distal to the coronary stenosis is 70 mm Hg, the FFR is 0.70 (Fig. 1). FFR values lower than 0.75 were found to correspond well with non-invasive measures of myocardial ischaemia [10]. This 0.75 FFR threshold was evaluated in patients with stable ischaemic heart disease in the DEFER study, where deferral of percutaneous coronary intervention (PCI) in coronary stenosis with an FFR value of >0.75 was not associated with increased rates of adverse events compared with PCI in this lesion subset [11–13]. Therefore, the results of the DEFER study were of particular importance at the time, because the FFR gave interventional cardiologists a quantifiable method to counteract the ‘oculo-stenotic reflex’. The randomised Fractional Flow Reserve versus Angiography for Multivessel Evaluation (FAME) trials have since documented that FFR-guided coronary intervention using a 0.80 FFR cut-off value reduces the number of coronary revascularisations compared with angiography-guided coronary intervention in patients with multi-vessel coronary artery disease, while maintaining favourable clinical outcomes [14–17]. The use of FFR in patients with multivessel coronary artery disease leads to changes in management decisions compared with angiography-based decision-making in over 40% of patients [18], and was noted to decrease the number of patients considered high risk when functional stenosis severity was added to the Syntax score [19]. As a result, an FFR-guided revascularisation strategy for patients with stable coronary artery disease has since been endorsed by both European and American clinical practice guidelines [20, 21]. The use of FFR to guide revascularisation using coronary artery bypass graft surgery has conversely not shown to reduce the incidence of graft failure, nor to improve clinical outcomes, although it may lead to simpler surgical revascularisation procedures by reducing the number of bypass grafts placed [22, 23]. Although FFR values of non-culprit coronary arteries in acute coronary syndrome patients may change over time due to recovery of microvascular function [24], the use of FFR was also documented to provide clinical benefits for the management in acute ST-segment elevation myocardial infarction (STEMI) patients. FFR-guided complete revascularisation at the time of the primary PCI procedure reduces delayed revascularisation of the non-culprit vessel in patients with STEMI and multivessel disease and thus reduces the need for staged procedures [25]. Such an FFR-guided complete revascularisation approach was noted to be cost-effective compared with a culprit-vessel only revascularisation strategy [26]. Similar studies in non-ST-segment elevation acute coronary syndrome patients are on-going [27]. However, despite guideline recommendations and clinical data supporting the use of FFR in a broad spectrum of patients undergoing coronary angiography, FFR is only used in minority of patients undergoing angiography for stable coronary artery disease [28].

## Non-hyperaemic pressure indices as an alternative to FFR

**Fig. 2:**
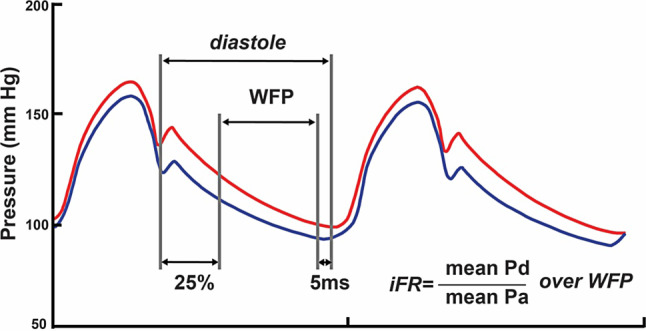
Instantaneous wave-free ratio. Instantaneous wave-free ratio (iFR) is defined as the mean distal coronary pressure (Pd) to mean aortic pressure (Pa) ratio over the wave-free period (WFP). The WFP is defined as starting 25% into cardiac diastole, and ending 5 ms before the end of diastole as illustrated

The limited adoption of FFR may partly be explained by the side effects associated with vasodilatory medication—which include dyspnoea, flushing and chest pain—as well as by the impact of additional diagnostic procedures on procedural time. The instantaneous wave-free ratio (iFR) was proposed as a vasodilator-free alternative coronary pressure ratio [29]. iFR is calculated as the ratio between distal coronary pressure and aortic pressure during the ‘wave-free period’, which starts one third of the way into diastole and ends 5 milliseconds before the start of systole (Fig. 2). Because the iFR is measured during the resting state, it does not require the use of vasodilatory medication. Both iFR and FFR possess equivalent diagnostic accuracy to identify myocardial ischaemia as defined by the gold standard of myocardial blood flow: [^15^O]H_2_O positron emission tomography perfusion imaging (PET) [30]. iFR was documented to lead to similar changes in treatment strategy in patients with multivessel coronary artery disease compared with an angiography-based strategy as was previously documented for FFR [31]. Moreover, two large randomised clinical trials have shown that guidance of revascularisation based on iFR with a cut-off value of 0.89 to depict haemodynamically significant stenosis resulted in non-inferior clinical outcomes at 1‑year follow-up as compared with FFR-guided revascularisation [32, 33]. Following these results, the European Society of Cardiology issued a class 1A guideline recommendation for the use of iFR to guide coronary revascularisation [34]. The two randomised clinical trials investigating iFR also documented that procedural time is shortened with the use of iFR versus FFR, and patients experienced less adverse procedural symptoms when iFR was used. These characteristics enhance the applicability of iFR in multivessel coronary artery disease patients. In non-culprit coronary arteries of patients with acute coronary syndrome, the diagnostic accuracy of iFR was reported to be similar to that in patients with stable coronary artery disease. Conflicting results have been reported regarding the change in iFR from the acute setting of STEMI to repeat invasive assessment [24, 35]. In terms of clinical outcomes, a meta-analysis of the randomised iFR studies documented no differences between iFR-guided revascularisation and FFR-guided revascularisation in non-ST-segment elevation acute coronary syndrome patients [36]. Randomised clinical outcome studies using iFR-guided PCI in STEMI patients have not been published, although currently available data suggest similar benefits of iFR can be expected in this patient subset.

Following the data on iFR, renewed interest has emerged regarding the resting distal coronary to aortic pressure ratio (Pd/Pa). Pd/Pa was documented to provide equivalent diagnostic accuracy to identify inducible myocardial ischaemia on [^15^O]H_2_O‑PET as compared with FFR and iFR [30]. Moreover, long-term prognostic value of Pd/Pa after deferral of coronary intervention was similar to that of FFR [37]. More recently, two alternative non-hyperaemic pressure ratios were proposed: the diastolic pressure ratio (dPR)[38] and the resting full-cycle ratio (RFR) [39]. Non-randomised studies have documented that these new non-hyperaemic pressure ratios achieved high correlation coefficients with iFR, and have suggested similar prognostic value [40, 41]. However, clinical guidelines do not currently mention the use of these indices.

## Coronary flow (velocity) reserve

**Fig. 3:**
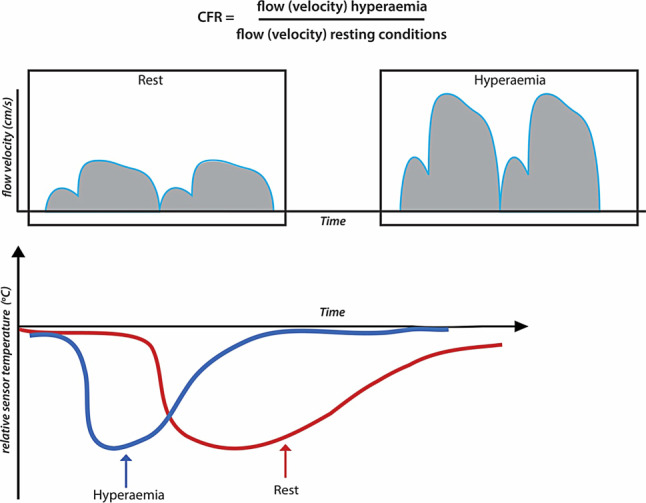
Coronary flow (velocity) reserve. Coronary flow (velocity) reserve (CFR) is defined as the ratio of hyperaemic to resting coronary flow (velocity). Coronary flow can be measured using the Doppler flow velocity technique (*upper panel*), and the coronary thermodilution technique (*lower panel*). The Doppler technique displays temporal changes in instantaneous peak coronary flow velocity, represented by the blue line in the schematic. The average peak coronary flow velocity over several cardiac cycles is used for the calculation of CFR. The thermodilution technique displays the individual thermodilution curves of a bolus of room-temperature saline. For thermodilution-derived flow measurements, the thermodilution curves are obtained in triplicate in resting and hyperaemic conditions. The mean transit time is calculated from these curves, and is average over three bolus injections in resting conditions, and three bolus injections in hyperaemic conditions for the calculation of CFR

**Fig. 4:**
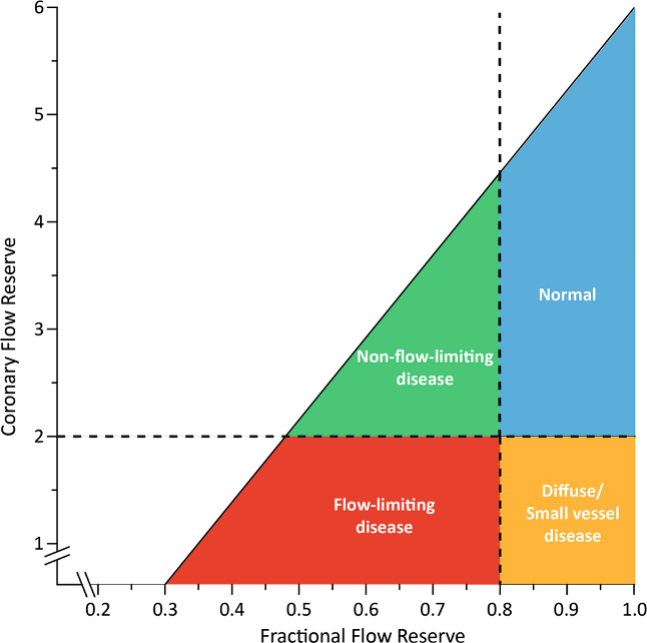
Conceptual plot of the fractional flow reserve (FFR)—coronary flow reserve (CFR) relationship. Four main quadrants can be identified by applying the clinically applicable cut-off values for FFR and CFR, indicated by the dotted lines. Patients in the upper right blue area are characterised by concordantly normal FFR and CFR, and patients in the red lower left area are characterised by concordantly abnormal FFR and CFR. Patients in the upper left green area and lower right orange area are characterised by discordant results between FFR and CFR, where the combination of an abnormal FFR and a normal CFR indicates predominant focal epicardial, but non-flow-limiting, coronary artery disease, and the combination of a normal FFR and an abnormal CFR indicates predominant microvascular or diffuse epicardial involvement in coronary artery disease. Adapted from Van de Hoef et al. [52] with permission of Wolters Kluwer Health

Coronary flow reserve (CFR) is defined as the ratio of maximal flow during vasodilated conditions, or hyperaemic coronary flow, to flow during conditions of coronary autoregulation, called resting or baseline coronary flow (Fig. 3). The concept of CFR therefore relates to the reserve capacity of the coronary circulation to accommodate to an increase in myocardial demand. The measurement of coronary flow reserve requires the use of specific sensor-equipped wires: either a Doppler sensor-equipped guide wire to obtain Doppler flow velocity measurements, or a guidewire equipped with a temperature-sensitive sensor to obtain coronary thermodilution-derived mean transit times. Of these, Doppler velocity measurements provide the most accurate assessment of true CFR values, even though Doppler measurements are considered more technically demanding [42]. The assessment of CFR necessitates measurement of coronary flow in both resting conditions and during coronary hyperaemia. A cut-off value of 2.0 is routinely used for CFR to delineate abnormal from normal CFR [1]. The diagnostic value of CFR for the identification of reversible perfusion deficits is similar to that of FFR [1, 43]. The prognostic value of CFR remains undisputed [44–49], but concerns remain regarding its sensitivity towards alterations in resting coronary flow, even though this impact is limited in large clinical studies [50]. Moreover, since CFR is impacted by both the epicardial and microvascular compartment of the coronary circulation, impairment of flow due to pathology in either of these compartments may result in abnormal CFR values which may limit the identification of pure stenosis-induced flow abnormalities. Nonetheless, selective evaluation of an intermediate lesion using CFR or FFR allows more adequate risk stratification and is more cost-effective than myocardial perfusion scintigraphy in patients with multivessel disease [48, 49, 51]. More recently, CFR has been applied in combination with FFR, which has led to the identification of typical FFR-CFR patterns that relate to basic coronary pathophysiology and physiology, as shown in Fig. 4 [52, 53]. Hence, combined assessment of CFR and FFR may allow more accurate identification of the underlying pathophysiology of chest pain syndromes [54]. These patterns have been documented to impact clinical outcomes in retrospective analyses [52, 55]. The DEFINE FLOW trial is now evaluating the prognostic value of combined CFR-FFR measurement for clinical decision-making in a prospective multi-centre setting [56]. Besides the setting of obstructive coronary disease, there is distinct interest in CFR as a marker of disease in patients with chest pain syndromes and no obstructive epicardial coronary artery disease. This setting is described in detail by Konst et al. elsewhere in this issue of the journal [57].

**Fig. 5:**
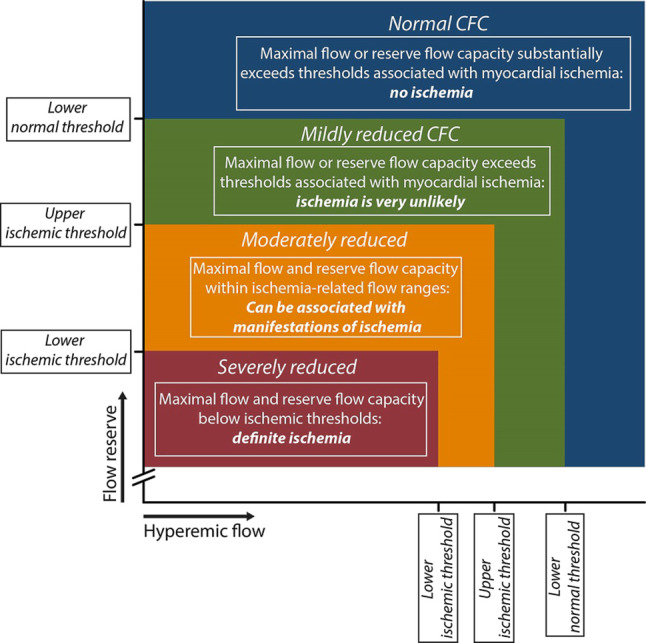
Coronary flow capacity concept. Since coronary flow reserve (CFR) equals hyperaemic to baseline average peak flow velocity (hAPV), a 2-dimensional map of CFR versus hAPV comprehensively describes the invasive flow characteristics of the coronary vasculature under investigation. Within this concept, four clinically meaningful categories are defined (coded with different colours in the graph) based on well-validated invasive CFR cut-off values and the corresponding hAPV percentiles. Reproduced from Van de Hoef, et al. [58] with permission of Elsevier

Following the remaining concerns on the sensitivity of CFR to resting flow conditions, a novel concept was introduced termed coronary flow capacity (Fig. 5; [55, 58–60]). This concept assumes that myocardial ischaemia is unlikely in settings where the vasodilator reserve (CFR) is normal, or where maximal blood flow is normal, and that myocardial ischaemia is likely in settings where both vasodilator reserve and maximal flow are severely reduced. This concept was documented to be less sensitive to clinical characteristics known to impact CFR [50], and was also found to provide enhanced risk stratification in patients with stable ischaemic heart disease over the use of CFR alone regardless of the method used to measure coronary flow [55, 58].

**Fig. 6:**
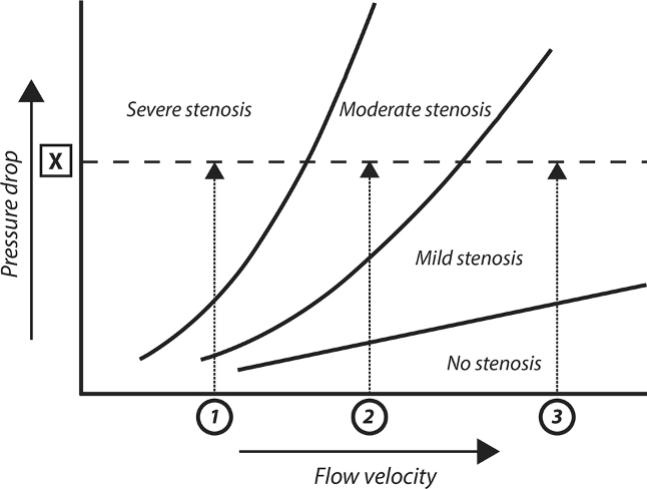
Stenosis pressure drop—flow velocity relationship. The stenosis-specific pressure-drop flow velocity relationship implicates that the pressure drop across a stenosis increases with increasing flow through the stenosis. Hence, a given pressure drop across a stenosis, *X*, may represent a stenosis severity ranging from mild to severe, depending on the flow velocity at which it was obtained, 1 to 3. The stenosis resistance index, defined as the ratio of the pressure drop across the stenosis to distal coronary flow velocity, ‘normalises’ the pressure drop for the magnitude of flow at which it was obtained, providing a more objective assessment of haemodynamic stenosis severity, and allows the attribution of the measured pressure drop to stenosis severity 1, 2, or 3

## Hyperaemic stenosis resistance index

The resistance to coronary blood flow induced by a stenosis can be calculated as the pressure loss across the stenosis divided by distal coronary flow velocity [61]. Since the pressure drop across a stenosis and distal coronary flow change in the same direction when maximal vasodilation is not achieved, hyperaemic stenosis resistance (HSR) as an index is relatively independent of the amount of hyperaemia achieved. Moreover, the benefit of such a resistance measurement is that it ‘normalises’ the pressure drop induced by the stenosis for the flow at which it was obtained (Fig. 6). HSR has only been defined using Doppler flow velocity measurements. Meuwissen et al. compared the diagnostic efficacy of HSR to both FFR and CFR using myocardial perfusion scintigraphy as the reference standard, where HSR demonstrated a superior diagnostic efficiency for non-invasively identified perfusion deficits. In the same study, a deferral threshold of <0.80 mm Hg/cm/s was established [60]. Early evaluation of its prognostic value by the same authors documented a high discriminatory value of HSR for future events, particularly in cases where discrepancy occurred between FFR and CFR [62].

## Basal stenosis resistance index

Since the stenosis resistance index is by definition relatively independent of the amount of hyperaemia induced, its assessment during resting conditions also allows to determine stenosis severity. This basal stenosis resistance (BSR) index was documented to provide equivalent diagnostic efficiency for the identification of perfusion deficits on myocardial perfusion scintigraphy compared with FFR [63]. Moreover, when contemporary dual sensor-equipped guide wires are used for its assessment, its discriminatory value closely approaches that of its hyperaemic counterpart, HSR [64]. In several studies, a deferral threshold of <0.66 mm Hg/cm/s has been defined for BSR [63–65], but no prognostic data have been published to date.

## Hyperaemic microvascular resistance index and index of microcirculatory resistance

Similar to the resistance induced by stenosis, the resistance of the microcirculation can be calculated as the distal coronary pressure divided by distal coronary flow [66, 67]. This calculation assumes that there is complete pressure loss across the coronary resistance vessels, and therefore that venous back pressure is negligible. The minimal resistance in the coronary microcirculation determined during coronary vasodilation is considered an important marker for its functional status, and can be calculated using either Doppler flow velocity (hyperaemic microvascular resistance (HMR) index) or coronary thermodilution (index of microcirculatory resistance (IMR)). Coronary Doppler flow-derived HMR provides a more accurate reflection of microvascular status, even though Doppler measurements are more technically challenging [42, 68, 69]. Part of the diagnostic difference is likely due to the dependence of IMR on the size of the perfused myocardial bed, which is theoretically less important in the assessment of HMR [70]. These measures of minimal microvascular resistance are linked to clinical outcomes both in stable coronary artery disease and acute coronary syndromes [66, 71]. In the latter setting, minimal microvascular resistance is associated with the presence of microvascular injury, as well as infarct size [72, 73]. Similar to CFR, there is also distinct interest in HMR as a marker of disease in patients with chest pain syndromes and no obstructive epicardial coronary artery disease, as discussed by Konst et al. elsewhere in this issue [57].

## Resting microvascular resistance and resistance reserve

Besides the minimal resistance of the microcirculation assessed at maximal coronary vasodilation, it is increasingly recognised that the functional status of the microcirculation during resting conditions and its vasodilator function are clinically important parameters [74, 75]. Dysfunction of the autoregulatory mechanism leading to increased resting flow levels has been associated with long-term adverse outcomes [44, 45]. Similarly, the reserve vasodilator capacity, analogous to coronary flow reserve, is an important marker for the functional status of the microcirculation.

## Absolute hyperaemic flow measurements

The indicator-dilution theory allows to measure absolute flow in ml/min by coronary thermodilution. This technique, using the same console and guide wire used for standard coronary thermodilution measurements, applies the continuous infusion of room-temperature saline through an infusion catheter. With a known infusion speed, and constant blood volume between the thermistors, absolute blood flow in ml/min can be calculated from the change in temperature induced by the infusion of saline [76, 77]. A shortcoming of absolute flow measurements is the fact that normal or cut-off values are not yet available. Absolute flow (and absolute resistance) depend on the amount of perfused myocardial mass [70, 76]. Hence, absolute flow values require correction for the amount of perfused myocardial mass, of which an ad-hoc invasive measurement is currently only available for Doppler flow velocity measurements [78]. Using coronary computed tomography angiography to estimate perfused myocardial mass, absolute invasive flow measurements show a strong agreement with absolute perfusion and microvascular resistance measured by PET [79]. The requisite of continuous saline infusion, which induces hyperaemia to the same extent as adenosine, implies that this technology does not allow measurements of absolute resting flow or CFR [76].

## Conclusion/future directions

Invasive coronary physiology is a rapidly developing field. After the initial oculo-stenotic reflex in angiography-based coronary intervention, it is now becoming customary to base treatment decisions on coronary pressure measurements that relate to the haemodynamic significance of the stenosis. Yet, we increasingly recognise the limitations of a stenosis-centred approach, and scientific efforts regarding a comprehensive assessment of the coronary circulation using the combination of coronary pressure measurements and coronary flow measurements, or the derived stenosis and microvascular resistance indices, increasingly document the relevance of more complex coronary physiology for clinical decision-making. Considering that the latest ESC clinical practice guidelines now support these advanced coronary physiology tools for clinical decision-making, the future is likely to see enhanced incorporation of comprehensive coronary physiology strategies in daily clinical practice.
